# The genomics of major psychiatric disorders in a large pedigree from Northern Sweden

**DOI:** 10.1038/s41398-019-0414-9

**Published:** 2019-02-04

**Authors:** Jin Szatkiewicz, James J. Crowley, Annelie Nordin Adolfsson, Karolina A. Åberg, Maaike Alaerts, Giulio Genovese, Steven McCarroll, Jurgen Del-Favero, Rolf Adolfsson, Patrick F. Sullivan

**Affiliations:** 1 Department of Genetics, University of North Carolina at Chapel Hill, Chapel Hill, NC USA; 2 Department of Clinical Sciences and Psychiatry, University of Umeå, Umeå, Sweden; 3 Center for Biomarker Research and Precision Medicine, Virginia Commonwealth University, Richmond, VA USA; 4 Center of Medical Genetics, University of Antwerp and Antwerp University Hospital, Antwerp, Belgium; 5 Broad Institute of MIT and Harvard, Cambridge, MA USA; 6 VIB Center for Molecular Neurology, Universiteitsplein 1, Antwerp, Belgium and Multiplicom N.V., Galileilaan 18, Niel, Belgium; 7 Department of Psychiatry, University of North Carolina at Chapel Hill, Chapel Hill, NC USA; 8 Department of Medical Epidemiology and Biostatistics, Karolinska Institutet, Stockholm, Sweden

## Abstract

We searched for genetic causes of major psychiatric disorders (bipolar disorder, schizoaffective disorder, and schizophrenia) in a large, densely affected pedigree from Northern Sweden that originated with three pairs of founders born around 1650. We applied a systematic genomic approach to the pedigree via karyotyping (*N* = 9), genome-wide SNP arrays (*N* = 418), whole-exome sequencing (*N* = 26), and whole-genome sequencing (*N* = 10). Comprehensive analysis did not identify plausible variants of strong effect. Rather, pedigree cases had significantly higher genetic risk scores compared to pedigree and community controls.

## Introduction

The genetic basis of major psychiatric disorders (i.e., bipolar disorder, schizoaffective disorder, and schizophrenia) has been intensively investigated over the past decade atop of substantial efforts dating to 1980s^1^. Most of the implicated findings to date have been for common genetic variants^1–4^, with significant common-variant genetic correlations between these disorders^5^. Although these results are unquestionably informative^6^, they usually implicate broad genomic regions with imprecise connections to particular genes. This complicates the design of subsequent neuroscience experiments of which major tools are optimal for the evaluation of single genes.

An experimental design that has been insufficiently exploited in psychiatry genetics in the current era is the comprehensive study of large, densely affected pedigrees^1^. An optimal strategy would apply assays that cover the most likely types of genetic variation coupled with control of multiple comparisons to enhance reproducibility. Although dense pedigrees can result from high common-variant genetic burden^7^, they can also be explained by rare variation of strong effect inherited from common ancestors. Systematic searches of such pedigrees are a complementary strategy to attempt to identify genetic variants of strong effect.

**Fig. 1:**
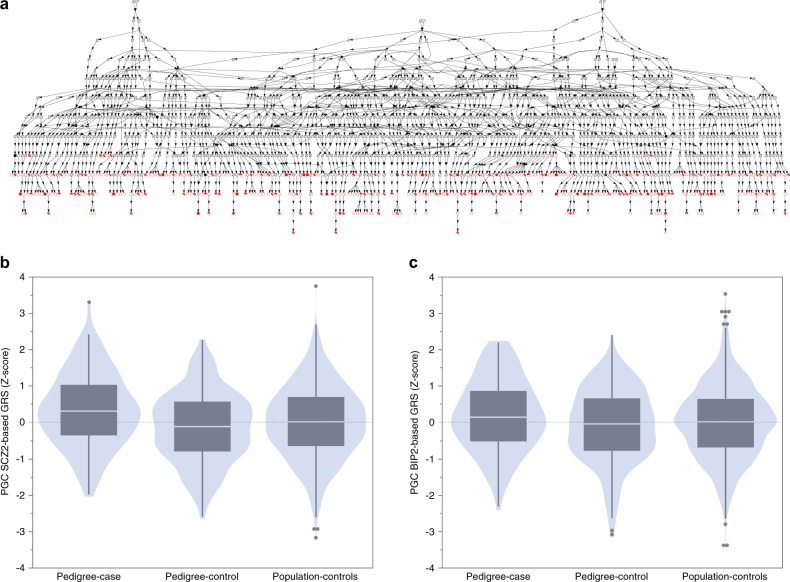
**a** Pedigree diagram of a fraction of the Northern Sweden pedigree. This demonstrates that all subjects in the study (at bottom, filled = affected, open = unaffected) have a complex pattern of inheritance. All connect to three founder pairs. **b** Genetic risk scores (GRS) for schizophrenia in pedigree cases (*N* = 128, narrow definition of illness), pedigree controls (*N* = 201), and population controls with no lifetime serious mental illness from Northern Sweden (*N* = 1267). Pedigree cases had significantly greater schizophrenia GRS than pedigree controls and population controls. Pedigree controls and population controls were similar. **c** As in Fig. 1b but showing GRS for bipolar disorder. GRS for bipolar disorder were slightly greater in pedigree cases than pedigree controls. The training sets for schizophrenia and bipolar disorder were from the PGC after removing Swedish studies. Shown are violin plots with overlaid outlier-style bar plots

Northern Sweden has a notably high prevalence of bipolar disorder and schizophrenia^8^ with a regional prevalence of major psychiatric disorders around eight times higher than Sweden as a whole^9^. There is a large pedigree in Northern Sweden of which origins (based on contemporaneous church records) date to three founder couples born around 1650. Individuals in this 14-generation pedigree (Fig. 1a) have increased lifetime risks of bipolar disorder, schizophrenia, and schizoaffective disorder. Due to its isolation and low migration rates, genetic evaluation of this pedigree may maximize chances of identifying rare genetic variation of strong effect inherited from common ancestors. Indeed, a genetic linkage study reported a heterogeneity LOD score of 5.05 (*P* = 0.00085) in a subset of the pedigree.

We applied a systematic genomic approach to the pedigree. Genomic assays included karyotyping (*N* = 9), genome-wide SNP arrays (*N* = 418), whole-exome sequencing (*N* = 26), and whole-genome sequencing (*N* = 10). Our goal was to identify a genetic contribution to the unusually high prevalence of major psychiatric disorders in this multiplex pedigree. Potential explanations range from a genetic variant of strong effect inherited from a common ancestor (prevalent in this pedigree but otherwise very rare) to an unusually high burden of common variants that individually have weak effects (i.e., high genetic risk scores, GRS). The latter possibility has been suggested by theory^10^ and demonstrated empirically for bipolar disorder^7^.

## Methods

### Subjects

This Northern Swedish pedigree (Fig. 1a) is from a geographically isolated region near the Arctic Circle. Prominent European settlement of this region dates to the early 1300 s with low rates of immigration and emigration. Births and marriages were systematically recorded in parish registers (URLs). Based on genealogical and hospital registers, a large number of patients with major psychiatric disorders were identified who share ancestry with three founder couples born around 1650. Construction of the pedigree using a top-down and bottom-up approach resulted in a 14-generation pedigree containing over 21,000 individuals. Lifetime psychiatric diagnoses were established using hospital register data, psychiatric clinical records, and structured diagnostic interviews administered by senior research psychiatrists and nurses^11,12^. Controls had no lifetime history of mental illness. The narrow definition of affection included subjects with lifetime diagnoses of bipolar 1 disorder, schizoaffective disorder, and schizophrenia. The broad definition of affection expanded the narrow definition by adding bipolar 2 disorder and bipolar disorder not otherwise specified (NOS). All participants provided written informed consent for participation in the study.

### Genomic assays

Table 1a summarizes the assays applied to individuals from the large Northern Swedish pedigee.

**Table 1a:** Technologies applied to the dense Swedish pedigree

Method	Sample	Technology and genetic variation assessed
Karyotyping	*N* = 9 (7 BIP1, 2 SCZ)	Standard Giemsa banding; large, “microscopic” structural variants
Pedigree linkage analysis	*N* = 378 (152 BIP, SCZ, or SAD; 226 unaffected relatives)	472 short tandem repeat markers; regions shared IBD
SNP genotyping	*N* = 418 (81 BIP1, 67 BIP2, 22 BIP-NOS, 18 SCZ, 29 SAD; 201 controls	Illumina OmniExpress SNPs, CNVs
Whole exome sequencing	*N* = 26 (12 BIP1, 13 BIP2, 1 SAD)	Agilent SureSelect, Illumina HiSeq; exonic SNVs, indels
Whole genome sequencing	*N* = 10 BIP1	Illumina X Ten; SNVs, indels, structural variants, trinucleotide repeats

*Karyotyping* was performed on fresh venous blood samples using standard methods in a hospital-based clinical genetics unit. The goal was to evaluate “microscopic” structural variants types of which can be missed by other methods.

*Microsatellite markers*: For linkage analysis, 472 short tandem repeat markers were genotyped (Alaerts et al., unpublished thesis), 411 markers with a mean inter-marker distance of 7.6 cM, and 61 additional markers for fine-mapping in eight chromosomal regions. PCR amplifications were performed under standard reaction conditions using fluorescent-labeled primers. PCR-products were sized on an ABI 3730 Sequencer (Applied Biosystems) and genotypes were scored using TracI. Subjects included affected individuals (*N* = 152 with schizophrenia, schizoaffective disorder, or bipolar disorder) and their unaffected first-degree relatives (*N* = 226). Linkage regions were used to prioritize genomic regions for evaluation.

*SNP genotyping* was done using OmniExpress arrays (Illumina, San Diego, CA, USA), and genotypes were called using BeadStudio (Illumina). Quality control filters excluded SNPs that had missingness ≥0.05, MAF <0.01, >2 Mendelian errors in trios, or violated Hardy-Weinberg Equilibrium (*P*_HWE_ < 1 x 10^−6^) in 201 unaffected individuals from the pedigree. A total of 621,639 SNPs passed quality control. Subject quality control filters excluded one subject with missingness >0.03, and one member in each of two monozygotic twin pairs were removed. These data were used to identify large structural variants, to estimate GRS, and to prioritize genomic regions for evaluation.

*Array-based structural variation*: As detailed elsewhere^13^, we evaluated autosomal structural variation using PennCNV^14^, and removed lower confidence calls (size < 50 kb, <10 probes, confidence score < 10) or structural variants mostly overlapping a gap in the reference genome or a region subject to recombination in peripheral white blood cells. One sample with aberrant log-R ratio was removed. Neighboring structural variants were merged. Case-control studies often focus on uncommon or rare structural variation (e.g., frequency <0.01 in controls). We did not apply this filter to this pedigree as not to exclude a potentially salient structural variant carried by multiple cases.

*Whole-exome sequencing (WES)* used exome capture (Agilent SureSelect Human All Exon v.2 Kit, targeting 189.9 K genomic regions, 32.76 Mb), followed by WES using Illumina HiSeq 2000 and HiSeq 2500 instruments (76 bp, paired-end). We used the Genome Analysis Toolkit (GATK) Version 2 Best Practices pipeline for preprocessing and variant discovery. Key steps for preprocessing included read alignment to hg19 using bwa-mem, removal of duplicated reads, local realignment around indels, and base quality recalibration, which result in analysis-ready .bam files. Key steps for variant discovery included variant calling using HaplotypeCaller (per-sample) to generate single-sample gVCFs, joint genotyping calling on the gVCFs for all samples, and variant recalibration to assign a well-calibrated probability to each variant call. The result is a VCF file for all samples, based on which we retained only the PASS variants and separated SNPs from indels. Quality control of the .bam files was done using Picard tools to evaluate the distributions of quality metrics. Quality control of VCF files was done using the “CollectVariantCallMetrics” tool in Picard to evaluate the distribution of SNV quality metrics. SNP genotype concordance was >0.988 for all samples based on 23,783 overlapping SNPs between WES and OmniExpress arrays, indicating high reliability for PASS SNP calls. These data were used to evaluate whether rare, protein-altering exon variants were plausibly causal in the pedigree.

*Whole-genome sequencing (WGS)*: We selected 10 affected individuals with bipolar disorder from across the pedigree for WGS. These subjects did not carry a large CNV and were more distantly related (all pairwise $\hat \pi$ < 0.125). WGS used the Illumina HiSeq X Ten system with paired-end 150 base pair reads using PCR-free procedures. All procedures were per the manufacturers’ protocols, and we followed the GATK Best Practices pipeline for preprocessing and variant discovery. SNP genotype concordance was >0.998 for all samples (based on >320 K overlapping SNPs between WGS and OmniExpress arrays), indicating high reliability for the PASS SNP calls. First, we used Delly (v0.7.3, default parameters) to detect, genotype, and filter structural variants (deletions, tandem duplications, and inversions ≥500 bp.) Delly uses paired-end mapping signature and split-read refinement to discover structural variant sites. After QC and removal of variant sites with ≥50% reciprocal overlap with known deletion/duplication/inversion variants in the 1000 Genomes Project Phase 3 database, there were 670 deletions (median size 975 bp), 320 duplications (median size 1066 bp), and 395 inversions (median size 20.1 kb). We prioritized those shared among ≥8 individuals for further evaluation. Second, we used ExpansionHunter^15^ to search for pathogenic trinucleotide repeats (e.g., the CAG repeat in exon 1 of *HTT* that causes Huntington’s Disease). These data were used as a means to identify types of variation that would have been missed by other technologies.

### Identity-by-descent (IBD)

We searched for genomic regions inherited IBD from a common ancestor. First, we used genetic linkage analysis based on 472 polymorphic short tandem repeat markers genotyped in 152 narrow-affected and 226 unaffected individuals (Alaerts et al., unpublished thesis; this sample only partly overlaps with the 418 subjects with GWA, WES, or WGS). As linkage analyses on the complete pedigree were intractable, the pedigree was split in sub-pedigrees (maximum 400 bits). Multipoint nonparametric and parametric linkage analysis were done using Simwalk2 (v2.91)^16^ under an affected-only model with unaffected relatives considered “unknown”. Linkage results were converted to hg19 coordinates by mapping microsatellite probe sequences. Second, we applied BEAGLE (v4.1, default parameters)^17^ to genome-wide SNP data to estimate pairwise IBD segments in all genotyped individuals (broad affection definition versus controls and narrow versus controls). We applied DASH (v1.1.0, default parameters and a minimum cluster size of *N* = 10)^18^ to the pairwise IBD segments to infer haplotype clusters of IBD individuals. Due to the relatedness of subjects in the pedigree, we used GCTA (v1.26.0)^19,20^ to perform linear mixed model association testing. GCTA analyses had case-control status as the dependent variable, haplotype clusters as markers, and included the genetic relationship matrix estimated from autosomal genome-wide SNPs.

### Common variant analyses

We evaluated whether selected genomic regions could be linked to GWAS results for bipolar disorder^21^ or schizophrenia^22^ using partitioned LD score regression (pLDSC)^23,24^. pLDSC is an extension of LD score regression allowing estimation of whether a set of genomic regions (e.g., regions shared IBD) are enriched for the SNP-heritability of a trait based on GWAS summary statistics. We followed the authors’ recommendations in order to add our regions of interest to the pLDSC baseline model^23^. Significance was assessed using the enrichment *P*-value.

We computed GRS for bipolar disorder^21^ and schizophrenia^22^ using best practice procedures^22,25,26^. We contrasted cases and controls from the pedigree with a population-based control sample from the same region of Sweden^22^. All three groups were genotyped with Illumina OmniExpress arrays. To create training sets, we obtained GWAS summary statistics (***URLs***) and used METAL^27^ to redo meta-analysis of case-control data from European samples after excluding Swedish studies^26^. These training sets were used to select SNPs and weight risk alleles for GRS calculation (variants were excluded if not biallelic, lacking dbSNP rsID, strand-ambiguous, MAF < 0.05 or >0.95, or INFO < 0.8). To select relatively independent SNPs for GRS calculation, we applied LD-clumping (*r*^*2*^ *<* 0.1 in 1 Mb windows) using 1000 Genomes Project European samples as the LD reference.

## Results

### Pedigee description

Figure 1a depicts a portion of the pedigree (N = 418 individuals in the bottom generations), and Table 1b summarizes their clinical and demographic features. These individuals had lifetime diagnoses of bipolar 1 disorder (*N* = 81), schizoaffective disorder (*N* = 29), schizophrenia (*N* = 18), bipolar 2 disorder (*N* = 67), bipolar disorder NOS (*N* = 22), or no lifetime history of a serious psychiatric disorder (*N* = 201). There were 244 females (58.4%) and 174 males (41.6%). The narrow definition of illness included bipolar 1 disorder, schizophrenia, and schizoaffective disorder (128 cases and 201 controls), and the broad definition added bipolar 2 disorder and bipolar disorder NOS (217 cases and 201 controls). The mean age (standard deviation) at participation was 56.3 (16.2) years for cases and 60.0 (15.5) years for controls; most subjects had traversed much of the period of lifetime risk for these disorders. Genomic assays were done on *N* = 418 individuals, including SNP arrays (*N* = 418), WES (*N* = 26), WGS (*N* = 10), and karyotyping (*N* = 9). Of the 418 subjects, 300 individuals were part of 72 first-degree relative clusters. Evaluation of genome-wide SNPs confirmed expected relationships. As anticipated, many pedigree members had low levels of relatedness (Figure S1a-c).

**Table 1b:** Clinical and demographic summary

Diagnosis	*N*	Age at onset	Age at sampling	Percent male (*N*)
BIP1	81	26.4 (13.4)	59.3 (14.5)	40.7% (33)
BIP2	67	28.3 (16.3)	52.0 (18.5)	32.8% (22)
BIP-NOS	22	28.2 (16.3)	62.6 (18.7)	27.3% (6)
SAD	29	20.4 (7.4)	50.7 (10.6)	51.7% (15)
SCZ	18	23.8 (7.3)	59.5 (13.3)	72.2% (13)
Pedigree controls	201	NA	60.0 (15.5)	42.3% (85)

*BIP1* bipolar disorder type I, *BIP2* bipolar disorder II*, BIP-NOS* bipolar disorder not otherwise specified, *SCZ* schizophrenia*, SAD* schizoaffective disorder, *IBD* identical-by-descent, *SNP* single nucleotide polymorphism, *SNV* single nucleotide variant, *CNV* copy number variant, *Indel* insertion/deletion polymorphism, *NA* not applicable

### Context

To better understand this pedigree, we analyzed genome-wide SNP data from multiple global samples as well as the pedigree. Using ancestry principal components analysis, we found that subjects from the Northern Sweden pedigree clustered with other European-ancestry samples on the world ancestry map (Figure S2). Within European samples, subjects from the Northern Swedish pedigree were at the extreme end of a north–south Swedish cline (PC1) that was different from Finnish ancestry (PC2) (Figure S3, consistent with our prior report^28^). People born in the northermost counties of Sweden had features consistent with a genetic isolate, and this was particularly pronounced in the Northern Swedish pedigree. Members of the pedigree had high levels of runs of homozygosity and genome-wide SNP homozygosity similar to or greater than that found in Finland (Figure S4). For example, the mean sizes of lengths of genome-wide runs of homozygosity per subject were 29.1 Mb in the Northern pedigree, 24.7 Mb in subjects from other studies born in the two Northernmost Swedish counties, 16.1 Mb in Finnish subjects from the 1000 Genomes Project, 7.9 Mb in southern Sweden, and 3.5 Mb in northwest Europeans. This suggests the particular utility of this large Northern Swedish pedigree in identifying genomic variants conferring relatively high risk for major psychiatric disorders.

### Structural variation

Structural variants inherited from a common ancestor are a plausible type of causal genetic variant. We begin with this hypothesis as structural variation is an obvious potential cause for psychiatric disorders in a dense pedigree, and the presence of such a structural variant would impact all subsequent analyses.

First, as “microscopic” structural variants may be missed by array-based assays of structural variation (e.g., balanced translocations), we performed clinical karyotyping of nine individuals from across the branches of the pedigree (seven bipolar disorder, two schizophrenia, six males, three females). All had normal karyotypes suggesting that a microscopic structural variant was unlikely to explain inheritance of major psychiatric disorders in this pedigree (Figure S5a).

Second, we evaluated structural variants detectable with SNP arrays, beginning with structural variants previously associated with serious psychiatric disorders (Figure S5b)^29–31^. We identified three cases with three different structural variants (Figure S5c): (a) a man with Bipolar 2 disorder and a 22q11.21 2.5 Mb duplication; (b) a woman with Bipolar 2 disorder and a 16p11.2 603 kb deletion; and (c) a man with schizoaffective disorder and a 16p13.11 1.02 Mb duplication. We also searched but did not identify any recurrent CNVs >50 kb that were significantly more common in cases (Figure S6a-b). We evaluated structural variant burden^32^ using standard approaches^13^ but found no significant differences between cases and controls.

Third, to ensure that we were not missing structural variants below detection thresholds for karyotyping and SNP arrays, we conducted whole-genome sequencing (30x coverage) of 10 cases (all with bipolar 1 disorder) from across the pedigree. We manually inspected all CNVs called in ≥8 of 10 cases and not found in the 1000 Genomes Project (phase 3). This included 115 deletions (median size 1.1 kb), 38 duplications (median size 961 bp), and 72 inversions (median size 85.8 kb). Most of these structural variants overlapped segmental duplications or gene-poor regions of the genome and were likely false positives or inconsequential. The genes that had coding regions affected were *HLA-DRB5* (deletion and duplication), *PRR21* (deletion), *HLA-A* (duplication), and *SMC1B* (duplication). No inversion breakpoints disrupted coding regions of any gene. Overall, we identified no structural variants suspected to account for the elevated rate of psychosis in this pedigree.

Using the PCR-free whole-genome sequencing data from 10 cases, we searched for known pathogenic trinucleotide repeat polymorphisms (e.g., *C9orf72* and frontotemporal dementia) using ExpansionHunter along with a similar in-house approach (that previously identified a pathogenic Huntington’s disease CAG-repeat expansion^33^). None were identified.

Thus, we found no indications of known or novel structural variants that might explain inheritance of major psychiatric disorders in this Northern Swedish pedigree.

### Regions shared IBD from common ancestors

This multiplex pedigree originated from a few shared ancestors and has features of a genetic isolate. It is thus possible that cases in the pedigree inherited genetic risk factors IBD, and such regions might be sufficiently small as to facilitate single gene identification. We identified putative IBD regions using complementary approaches: linkage analysis (Figure S7) and estimation of pairwise IBD segments followed by inferring haplotype clusters of IBD individuals (using BEAGLE and DASH).

The linkage regions (chr2:85.286-124.629 Mb, chr8:77.835-100.669 Mb, and chr11:4.735–23.119 Mb) were unremarkable. Bioinformatic analysis using GREAT^34^ showed no functional enrichments (i.e., Gene Ontology molecular function, biological process, and cellular compartment). These regions contained 2.58% of the SNPs used by pLDSC but were not enriched for the SNP-heritability of bipolar disorder (enrichment 0.980, SE 0.152, *P* = 0.89) or major depression (enrichment 0.952, SE 0.173, *P* = 0.78). Contrary to expectations, the SNP-heritability of schizophrenia was significantly depleted in these regions (enrichment 0.652, SE 0.100, *P* = 6.8e-4).

DASH identified many regions-shared IBD by small clusters of subjects. We focused on those with nominal significance between cases and controls (*P* < 0.01). Bioinformatic analysis using GREAT^34^ showed no enrichments for any annotation. These regions were not enriched for SNP-heritability of bipolar disorder (enrichment 0.978, SE 0.029, *P* = 0.45), schizophrenia (enrichment 1.012, SE 0.023, *P* = 0.59), or major depression (enrichment 1.00, SE 0.0278, *P* = 0.92). Analysis of regions with case-control difference at *P* < 0.1 showed similar results. Statistical analysis of these regions using the broad and narrow definitions of illness showed no significant findings (Figure S8).

### Exonic variation

We evaluated WES data from 26 cases from across the pedigree (12 bipolar 1 disorder, 1 schizoaffective disorder, and 13 bipolar 2 disorder), and identified 129,651 variants (1.4% novel). We annotated variants passing quality control using the Variant Effect Predictor^35^. The intent of this analysis was to identify exon variants that might plausibly be causal in this pedigree. We selected variants that were: (a) either unknown or had a maximum allele frequency in any 1000 Genomes Project or GnomAD cohort <1e-5;^36^ (b) had predicted “high” impact or were located in a conserved coding region ( >ninetieth percentile);^37^ and (c) located in a brain-expressed gene (14 K genes defined as expression >1 in any GTEx brain sample^38^). This yielded 102 variants. Manual review of this list did not yield any high-confidence predictions that were present in more than half of the cases.

### Burden of common variation using GRS

We evaluated the contribution of common variation in the Northern Swedish pedigree using GRS. The training sets for schizophrenia and bipolar disorder were from the PGC after removing Swedish studies^21,22^. We contrasted three groups: cases from the pedigree (*N* = 128, narrow definition of illness), controls from the pedigree (*N* = 201), and an independent set of controls with no lifetime serious mental illness from Northern Sweden (*N* = 1267). The schizophrenia GRS data are depicted in Fig. 1b. The mean GRS were significantly different between groups (*F*_2,1593_ = 9.06, *P* = 1.22e-4). Post-hoc mean testing using Tukey-Kramer HSD showed that pedigree cases had significantly greater schizophrenia GRS than pedigree controls (difference = 0.474, *P* = 8.2e-5) and population controls (difference = 0.330, *P* = 0.0011), but that pedigree controls were similar to population controls (difference = 0.143, *P* = 0.14). Figure 1c shows the bipolar disorder GRS. The mean GRS were marginally different between groups (*F*_2,1593_ = 3.39, *P* = 0.034). Post-hoc tests showed that pedigree cases have slightly greater bipolar GRS than pedigree controls (mean difference 0.294, *P* = 0.026), but that the other two comparisons were not significantly different (pedigree cases-population controls, *P* = 0.10 and pedigree controls-population controls, *P* = 0.36). These results provide a potential explanation for the clustering of major psychiatric illnesses within the Northern Swedish pedigree.

## Discussion

Densely affected pedigrees can result from several genetic processes. The two extreme possibilities are that affected individuals inherit single variants of strong effect from common ancestors or that they inherit unusually high numbers of common variants of subtle effect. Our comprehensive analyses of many potential types of rare variation did not yield any candidate loci of strong effect (i.e., via karyotyping, CNV, WES, and WGS analyses). Given that our sample has features of a genetic isolate, we may have maximized our chances for detecting such variants.

Our results were consistent with inherited burden of common variation given that pedigree cases had somewhat higher schizophrenia and bipolar GRS compared to pedigree and community controls. We observed stronger prediction from schizophrenia GRS than from bipolar GRS, which we attribute to the greater informativeness of the schizophrenia GRS training set. The finding of higher GRS in pedigree cases was anticipated from theory^7^ and from our prior study of a pedigree dense with bipolar disorder^7^. In this and in our prior study, the pedigrees were from geographically isolated areas with low migration rates. We speculate that founders had above average GRS and that subsequent matings with individuals from the same region served to increase GRS (on average). This process of “concentrating” GRS over generations could then increase the risk of affection in pedigree members. A compatible hypotheses was forwarded by de Jong et al. in a genetic study of a large pedigree dense with mood disorders^39^.

The genetic study of dense pedigrees is a logical complementary strategy for gene-finding in psychiatric genetics^40^. This was leveraged in Scandinavia to identify *RBM12* as a risk locus for psychosis^41^. Indeed, the PGC has scaled this approach: using its global networks, the PGC is in the process of ascertaining and genetically evaluating 100 densely affected pedigrees^1^. It is probable that most will result from concentrations of common variation but even one confident identification of a causal rare variant would be an important advance.

We note that we cannot exclude the presence of risk variants with more complex inheritance patterns, variants with cryptic functional effects, or variants missed by our genomic assays. However, the presence of many common-variant risk alleles in a pedigree is a potential explanation for a dense pedigree.

## Supplementary information

Supplemental Figures S1–S8
